# Keys for Action: An Efficient Keyframe-Based Approach for 3D Action Recognition Using a Deep Neural Network

**DOI:** 10.3390/s20082226

**Published:** 2020-04-15

**Authors:** Hashim Yasin, Mazhar Hussain, Andreas Weber

**Affiliations:** 1 Department of Computer Science, National University of Computer and Emerging Sciences, Islamabad 44000, Pakistan; mazhar.h@nu.edu.pk; 2 Department of Computer Science II, Universität Bonn, 53115 Bonn, Germany

**Keywords:** action recognition, deep neural network (DNN), motion capture (MoCap) datasets, keyframe extraction

## Abstract

In this paper, we propose a novel and efficient framework for 3D action recognition using a deep learning architecture. First, we develop a 3D normalized pose space that consists of only 3D normalized poses, which are generated by discarding translation and orientation information. From these poses, we extract joint features and employ them further in a Deep Neural Network (DNN) in order to learn the action model. The architecture of our DNN consists of two hidden layers with the sigmoid activation function and an output layer with the softmax function. Furthermore, we propose a keyframe extraction methodology through which, from a motion sequence of 3D frames, we efficiently extract the keyframes that contribute substantially to the performance of the action. In this way, we eliminate redundant frames and reduce the length of the motion. More precisely, we ultimately summarize the motion sequence, while preserving the original motion semantics. We only consider the remaining essential informative frames in the process of action recognition, and the proposed pipeline is sufficiently fast and robust as a result. Finally, we evaluate our proposed framework intensively on publicly available benchmark Motion Capture (MoCap) datasets, namely HDM05 and CMU. From our experiments, we reveal that our proposed scheme significantly outperforms other state-of-the-art approaches.

## 1. Introduction

Human action recognition and behavior analysis has been an active research area in recent decades because of its wide range of potential applications, including human–computer interaction applications, e.g., sport annotations and fitness training, game and film industries, computer animations, robotics, content-based data retrieval, health monitoring, and medical rehabilitation. For this reason, the demand for capturing and rendering 3D human motion is continually increasing. In general, studio-like environments with well-developed motion capture systems are used to capture the dynamic movements of an actor or object. The motion capture data basically represent human motions in the form of the spatiotemporal trajectories of the specified human skeleton joints [1]. The motivation behind motion capturing is to record the motion and then re-utilize it to perform different kinds of tasks rather than generate the motion synthetically. In order to capture human motion, a variety of sources are utilized, such as mechanical systems in which potentiometer and fiber optics are used; magnetic-/acoustic-based systems [2]; camera-based systems [3,4,5] in which expensive, high-speed and high-definition cameras are used; high-speed optical motion capture systems using photosensors [6]; inertial sensor- and accelerometer-based systems [7,8]; and hybrid systems [9]. As another domain, 3D motions are reconstructed from different sources, e.g., reconstruction from video or image data [10,11,12,13,14] and reconstruction from accelerometer data [15,16]. In short, motions captured or generated by different sources of means are abundant and contain a lot of hidden knowledge and information that may be exploited further in different types of applications, such as those mentioned above.

There exists a variety of action classification methods that are based on different input data; the input may be in the form of simple RGB videos or spatiotemporal joint trajectories acquired by means of a sensor system (e.g., mechanical, magnetic, optic, inertial, non-optic wearable sensors and RGB-D sensors, such as Kinect) or an estimated 3D skeleton from image data or a hybrid system. Although a lot of research has been done in the domain of action recognition, there still exist numerous challenges, such as viewpoint variations, different human body sizes and appearances, and illumination factors that may influence the efficiency and performance of existing algorithms [17]. Moreover, each performing actor has his or her own way and style of executing the same action. The actions may also have many variations in terms of speed and length. In addition to these inter-class variations, extensive intra-class variations make the task more difficult. For example, it is not an easy task to differentiate between *jogging* and *running*, *walkForward* and *walkBackward*, *sitDownChair* and *sitDownFloor*, *standUpSitChair* and *standUpSitFloor*, *rotateArmBackward* and *rotateArmForward*, etc. The example actions described in this paper are presented with the labels as it is used in [1,18,19]. In order to deal with all these challenges, a number of techniques have been proposed. One of these techniques is the skeleton-based approach, which has become very popular because it provides very comprehensive information as compared with approaches based upon 2D RGB images or depth sensor data [20]. It is comparatively more resilient to variations in illumination, occlusion factors, a complex and continuously changing background environment, etc. [21]. Skeleton-based features are more vigorous and robust as compared with depth-based features for both cross-subject as well as cross-view action recognition [17]. Action recognition based on 3D human skeleton joints is basically a problem of time-series analysis, where the action is recognized from sequences of articulated body poses over time [22,23,24]. Generally, the skeleton-based approach can be categorized into two major classes: (i) the human body posture comprising different body joints has been employed to recognize actions [20,25,26,27,28], and (ii) human body parts in the form of a cylinder have been utilized in the process of action classification [29,30,31]. In this context, this paper deals with the skeleton-based action recognition technique using human MoCap datasets, where the human body posture comprises different body joints.

**Figure 1:**
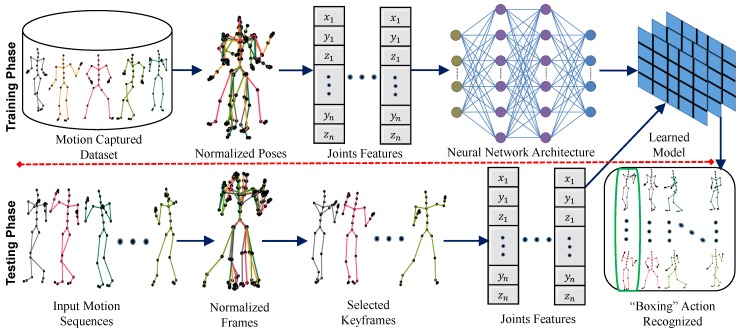
System overview. The first phase is the *training phase*, while the second phase represents the *testing phase*. In the *training phase*, 3D poses are normalized first by removing orientation and translational information from each pose, thus developing the normalized pose space. The joint features are extracted from these normalized poses and are given as input to the deep neural network in order to learn the model. The *testing phase* includes normalization, keyframe extraction and then the extraction of joint features, on the basis of which the action is recognized with the help of the learned model.

In this paper, we propose a novel and efficient approach for 3D action recognition based on the human body skeleton with 3D articulated joint trajectories. We first design a framework in order to learn the action model during the *training phase*, and we start by normalizing the sequence of poses to reduce the impact of orientation, as well as translation. Having obtained the normalized poses, we then successfully extract the joint features, which further contribute as input to the deep neural network in order to classify the 3D action in a supervised learning fashion. We empirically adjust the number of hidden layers and the number of neuron units within a hidden layer in order to come up with the final deep network architecture. We report all the particulars of experiments for the configuration of the deep neural network architecture in Section 4. We also propose and design a strategy that enables us to extract keyframes from a sequence of motion. As a result, we summarize the motion by discarding redundant frames and keep only the frames that have essential and comprehensive information about the action in the motion. The proposed keyframe extraction method reduces the latency, as the online system generally requires short latency for decision-making in human–machine interactions. In our experiments, we reveal and verify that a few informative keyframes, rather than all of the frames of a motion sequence, are sufficient to recognize the action accurately. The details about the proposed framework can be seen in Figure 1.

We evaluate our proposed approach thoroughly on popular benchmark MoCap datasets, namely HDM05 [32] and CMU [33]. We categorize these datasets further into four groups according to the motion classes, e.g., HDM05-65, HDM05-14, CMU-30, and CMU-14. We conducted several experiments in order to analyze our proposed scheme, and we describe them step by step. In particular, we analyze (i) the impact of essential keyframes on the overall process of action recognition in terms of accuracy and time complexity, (ii) the impact of the training MoCap datasets and, ultimately, and (iii) the impact of a variety of deep neural network architectures. We compare our approach with other existing state-of-the-art approaches and conclude that our proposed pipeline is not only computationally fast enough but also achieves comparatively better performance in terms of accuracy.

This paper is organized as follows: we first discuss the related methods and techniques in Section 2. In Section 3, we describe our proposed methodology step by step, including the process of normalization, the proposed keyframe extraction algorithm, and the details about the proposed deep neural network architecture. The experiments and the discussion on the obtained results are presented in Section 4. A comparison of our approach with other techniques is also available in Section 4. Finally, we conclude our work in Section 5.

## 2. Related Work

In this section, we discuss the background research work in the field of action recognition using motion capture datasets. There exists a variety of techniques to recognize 3D human action from a motion capture dataset. We categorize these techniques into two major classes, i.e., conventional machine-learning-based approaches and deep-learning-based algorithms.

### 2.1. Conventional Learning-Based Approaches

An abundance of research has been done on human action recognition based on conventional machine learning techniques, such as K Nearest Neighbor (KNN) classifiers [34,35], Support Vector Machine (SVM) [18,35,36,37,38,39,40], Hidden Markov Models (HMM) [41,42], clustering strategies [42,43,44], and Bayesian learning [45,46]. Most of these techniques first extract hand-crafted features [34,36,38,39,46,47] and then apply a learning algorithm in order to classify the action. Wu et al., in [34], propose action descriptors with a sliding temporal window of size 5, which includes joint position, angular velocity, and angular acceleration. They exploit three different modified K Nearest Neighbor (KNN) classifiers for the confidence of each frame, for the prediction of frame-wise labels, and, ultimately, for the final action classifications. In [18], Cho et al. propose a method that classifies the action while simultaneously reconstructing the given input motion sequences. This approach utilizes two sets of features: the first feature set consists of the relative positions of joints (PO) with temporal differences (TD), represented as (PO+TD); and the second set includes the relative positions of joints (PO) with temporal differences (TD) and normalized trajectories (NT) of motion, represented as (PO+TD+NT). A Hybrid Multi-Layer Perception with a deep autoencoder, a symmetric feedforward neural network, is then trained on this extracted feature set to perform the classification and reconstruction tasks simultaneously. The experiments were performed on the HDM05 MoCap dataset. For the evaluation, they also employed other classification techniques, such as Multi-Layer Perception (MLP), SVM, Extreme Learning Machines (ELM), and Hybrid Multi-Layer Perception (Hybrid MLP), with different learning rates λ={0,0.1,0.5,0.9}. Yang and Tian, in [46], propose a new action feature descriptor, e.g., eigenJoints, for action recognition, which is basically the combination of multiple action-related information types, such as the static posture of the actor, how the motion is performed, and the ultimate overall dynamics. For the selection of the most informative frame, they employ Accumulated Motion Energy (AME), which measures the dissimilarity between the frames. For the final action classification task, they deploy a non-parametric Naïve-Bayes-Nearest-Neighbor (NBNN) classifier. Vantigodi et al. [39] propose a method for action recognition that is based on two feature sets, the variance of skeleton joints and the time-weighted variance of the skeleton points, which incorporates the temporal information of the performed action. Both feature sets are embedded together for further model training. For action classification, a linear SVM and a correlation-based metric are employed. Liang et al. [42] introduce a local joint structure and 3D histogram-based local and global features in order to represent 3D actions. Linear Discriminant Analysis (LDA) is employed to reduce feature dimensionality, the k-means clustering algorithm is utilized to generate codewords, and Hidden Markov Models are deployed for action recognition on the basis of the codewords. Moussa et al. [38] propose a methodology that depends on high-level features that carry information about changes in human body dimensions during the performance of the action. Their proposed system comprises four stages: the extraction of skeleton details, parameter calculation, parameter encoding, and, finally, a classification module, in which a multi-class linear SVM classifier is employed.

Slama et al. [36] present a method in which 3D human skeleton motion is represented as geometric formulations, and an action is represented as a component of a Grassmann manifold. For classification, they employ a wrapped Gaussian model and a linear Support Vector Machine (SVM). Kovar and Gleicher, in their paper [47], propose a method to logically identify similar motion segments by employing a novel distance metric with which they numerically find similarity and closeness between motion segments from a large MoCap dataset. They further utilize these similar segments for automated motion registration, as well as for blending the continuous and parameterized space of 3D motions. Xiao and Song [45] propose a technique of Statistical Learning and Bayesian Fusion (SLBF) for motion clip similarity in which a motion feature database is developed by representative frames extracted through a fuzzy clustering strategy and gesture features. They basically combine category-based motion similarity distances and Canonical Correlation Analysis (CCA)-based distances through Bayesian estimation in order to find similar segments from a MoCap dataset. Kadu and Kuo [37] propose a multi-resolution string representation method using Tree Structure Vector Quantized (TSVQ) in order to generate a codeword for action classification. For final action recognition and classification, they considered a number of methods, such as (i) *Method A:* Motion-String Similarity including Sim-parameter with Level-*n* (*SLn*) and Max-parameter with Level-*n* (*MLn*), where n=10,11,12,13; (ii) *Method B:* a Pose-Histogram Classifier with Level-*n* (*SLn*), where n={3,4,5,6}; (iii) *Method C:* Two-Step Score Fusion with TSVQ; and (iv) *Method D:* Two-Step Support Vector Machine (SVM) Fusion with TSVQ. Overall, they achieved very good results with Two-Step (SVM) Fusion with the TSVQ method. Ko et al., in [43], deploy Principle Component Analysis (PCA) for dimensionality reduction, motion saliency and the k-means clustering algorithm in order to first extract informative and significant keyframes from human motion input sequences; then, they reconstruct these motion sequences to compare them with the input motion clip. In [48], Wu et al. employ a Self Organizing Map (SOM) and the Smith–Waterman algorithm to achieve efficient retrieval and, ultimately, indexing of the human motion capture data. For indexing, the SOM is utilized, while the local similarities between motion clips are computed with the help of the Smith–Waterman algorithm. They basically propose an unsupervised method for the indexing and clustering of motion clips, which are deployed further to classify the actions, as well. They enhanced their strategy of the motion map, as described in [44], where they present a cluster-based scheme for the indexing and retrieval of motion clips. They partition the human skeleton model into three body parts: the torso, the arms and the legs. They then measure temporal similarity information for each body part by the SOM and Smith–Waterman algorithm. In the end, a hierarchical clustering method is implemented to cluster similar data, as well as to find relationships between them. The authors of [41] propose a novel frame-by-frame action recognition approach by considering the algebraic velocity generated by different body parts of the 3D skeleton. For the classification of different action categories, a real-time Hidden Markov Model algorithm with Gaussian Mixture Models (GMM) is deployed. Barnachon et al. [49] propose an action recognition technique for ongoing action sequences. They compute the histogram of the action and then the Hausdorff distances accordingly, which are further warped by Dynamic Time Warping (DTW). For that purpose, they deploy dynamic programming in order to compute the final recognition score. Baumann et al. [50] propose an action graph, for which a kd tree is developed. The neighborhood of a query is fetched, which is further utilized in the action graph. Finally, action recognition is transformed into the shortest-path-finding problem, where the target is to find the shortest path through the action graph. This shortest path represents the final action. Laraba et al. [35] first represent 3D human motions as 2D RGB images, and then they employ classical machine learning algorithms, including KNN, SVM, Random Forest, and Convolutional Neural Network (CNN), for action classification. On the basis of their experiments, they claim that because the images of the motion sequences are represented in the RGB domain, the CNN outperforms all other competing techniques. Plenty of methods exist that employ key pose-based features or descriptors in order to classify actions [49,51,52,53].

### 2.2. Deep-Learning-Based Approaches

Hinton et al. [54] define a DNN as a neural network that consists of two or more hidden layers between the input layer and the output layer. The literature contains a number of techniques that employ deep-learning-based approaches; for example, [1,55,56,57] rely on a Convolutional Neural Network (CNN), [19,58,59,60] utilize a Recurrent Neural Network (RNN), as well as Long-Short Term Memory (LSTM), and [61] uses Deep Progress Reinforcement Learning (DPRL). Sedmidubsky et al. [1] propose a method for action recognition and segmentation in which the motions are mapped onto encoded RGB images. They first normalize the poses, and then the x-, y-, and z-coordinates of the poses are translated into red, blue, and green channels of the colors. They combine distance-based functions with a CNN classifier. In this way, they generate fixed-sized, highly descriptive feature vectors with 4096 dimensions. They learn motion characteristics by employing a CNN while performing indexing by a distance-based comparison. They further enhanced this approach towards the process of segmentation. Zhang et al. [59] extended an RNN model to the spatial domain by adding up simple geometric relational features that are based on the distances between skeleton joints. They use a three-layer-deep LSTM model in which they drop the in-cell connections. The geometric features are given as input to the first layer of the LSTM network, and the output of the first layer is provided as the input to the upper layer. A softmax layer is ultimately used on top of the highest LSTM layer. Liu et al. [55] propose a skepxel in which they combine spatial and spatiotemporal information in order to represent the skeleton joint sequences. Furthermore, they also add up relative joint velocities. In this way, the authors provide a more detailed hierarchical representation with micro-temporal relation and macro-temporal relation for learning through a CNN. They extended the Inception-ResNN CNN with their proposed scheme and obtained outstanding results. Tang et al. [61] recognize action by proposing Deep Progress Reinforcement Learning (DPRL) with a graph-based CNN. They extract the most informative frames from the input action video sequences through DPRL and employ a graph-based CNN in order to exploit the extrinsic, as well as intrinsic, human joint dependencies. Pham et al. [57] propose an SPMF (Skeleton Posture-Motion Feature) based on necessary spatiotemporal information extracted from skeleton poses and their motions in order to represent unique patterns that exist in skeletal movements. It is further enhanced by exploiting the Adaptive Histogram Equalization (AHE) method to build the action map. In the end, Deep CNNs (DCNN) based on the already-proposed DenseNet architecture are utilized for the purpose of final learning and action classification. In [19], the authors propose different Recurrent Neural Network (RNN) architectures for action recognition. Rather than use the whole skeleton, this approach divides the skeleton into five subparts (two legs, two arms, and one trunk) according to the skeleton structure in order to feed different recurrent network architectures, such as a Hierarchically Bidirectional Recurrent Neural Network (HBRNN-L) with Long-Short Term Memory (LSTM) architecture in the last network layer, a Hierarchically Unidirectional Recurrent Neural Network (HURNN-L) with LSTM, a Deep Bidirectional Recurrent Neural Network (DBRNN-L) with LSTM and Deep Unidirectional Recurrent Neural Network (DURNN-L) with LSTM, a Deep Bidirectional Recurrent Neural Network (DBRNN-T) with a *tanh* activation function, and a Deep Unidirectional Recurrent Neural Network (DBRNN-T) with a *tanh* activation function. Veeriah et al. [60] added a new gating strategy in LSTM to develop a differential RNN that depends on information obtained through the changes that occur in successive frames. Ijjina et al. [56] propose a fuzzy CNN to recognize action using human 3D skeleton data. They measure the temporal variation between the skeleton joints during action sequences and recognize local patterns.

## 3. Methodology

The detailed pipeline and framework of our proposed methodology can be seen in Figure 1. We discuss all the steps involved in our proposed methodology, one by one, as follows.

### 3.1. Normalization

**Figure 2:**
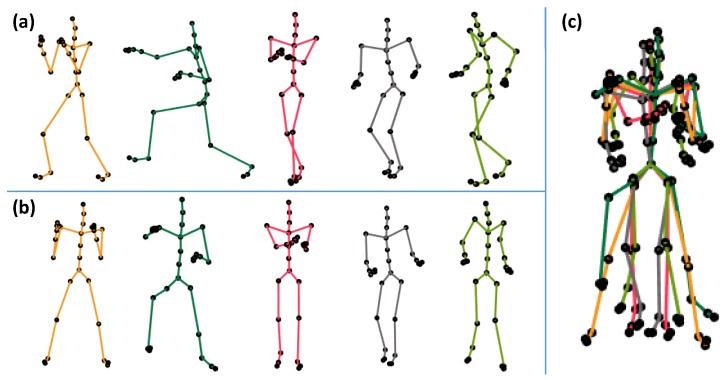
The process of normalization. (**a**) A representation of a few poses taken from different motion classes of the CMU-30 dataset. (**b**) The orientation information is removed from the poses. (**c**) The translational information is also removed to generate the final poses, which are ultimately normalized rotationally, as well as translationally.

The first step of the proposed pipeline is the process of normalization. We normalize each 3D pose *X* with 31 joints J that exist in the motion M. In fact, we eliminate the translational, as well as the orientation, information from the 3D pose so that we can avoid ambiguities and complexities that may arise because of such information. In the case of translational normalization, we translate the 3D pose in such a way that the pose must have its center of mass (the root joint) at the coordinates (0,0,0). We basically subtract the root joint coordinates from all other joint coordinates in order to shift the pose at the position (0,0,0). For orientation normalization, we rotate the joint trajectories along the *y*-axis (facing upward) so that the subject becomes just the frontal view: the skeleton faces towards the *x*-axis, and the hip joints are aligned to the *z*-axis. We first estimate the angle and then rotate all the joints of a pose with this angle about the *y*-axis. As a result, each pose has only the information about how the motion is performed, rather than where and from what viewpoint it is executed. An example of the normalization of different poses is shown in Figure 2.

### 3.2. Keyframes

In this paper, we propose a keyframe extraction technique to extract the most informative frames and to remove pose redundancy. As a result, human motion is effectively compressed and summarized. Moreover, in this way, we increase efficiency in terms of time and accuracy for action recognition, as well. In fact, the extraction of keyframes may be considered an indispensable step in an online recognition system that demands short latency for a quick response.

#### Implementation Details

Our proposed keyframe extraction strategy is iterative in nature. In each iteration, a new suitable keyframe is selected from the remaining frames of the input motion according to the similarity measure. For example, in the first iteration, we find *k* nearest neighbors of the first frame of the given input motion of size *n* within that input motion. In order to find the nearest neighbors in the J×3×n-dimensional space defined by the skeleton joints, we measure the average 3D Euclidean distance. We fix the size of $k=\frac{n}{2}$ so that the size of the nearest neighbors do not exceed the size of the motion. We further purify the nearest neighbors N in hand to select suitable candidate keyframes Φ by means of a threshold; a threshold is used to control the compression ratio for the number of frames that should be reduced. We report the details about the selection of the threshold in Section 4. To this end, we have candidate keyframes Φ from which we have to extract the final keyframes Ψ. We sort these candidate keyframes and then find the median frame, which is finally considered to be the keyframe. All the candidate keyframes are discarded from the input motion so that these frames do not participate again in subsequent iterations. The complete process is repeated until there are no input frames left. The details about the algorithm are presented in Algorithm 1.
**Algorithm 1** Keyframe Extraction Algorithm1:**procedure**KeyframesExtraction(M)2:   **inputs**: *$M=\{X_{1},X_{2},X_{3},\cdots ,X_{n}\}$, given input sequence of frames*3:   **persistent**: *n, total number of frames*   *k, total number of nearest neighbors*   *$N=\{{\hat{X}}_{1},{\hat{X}}_{2},{\hat{X}}_{3},\cdots ,{\hat{X}}_{k}\}$, retrieved nearest neighbors*   *$D=\{d_{1},d_{2},d_{3},\cdots ,d_{k}\}$, distances computed for each nearest neighbor*   *$\Phi =\{\phi_{1},\phi_{2},\phi_{3},\cdots ,\phi_{c}\}$, candidate keyframes*   *$\Psi =\{\psi_{1},\psi_{2},\psi_{3},\cdots ,\psi_{s}\}$, final selected keyframes*   *t, threshold value*4:   $k\leftarrow \frac{n}{2}$5:   s←16:   **while** (length(M)≠0) **do**7:    $X_{1}\leftarrow extractFrame\left(M\right)$8:    $\left[N,D\right]\leftarrow findNearestNeighbours\left(M,X_{1},k\right)$9:    i←110:    **while** (i<k) **do**11:     **if** $\left(d_{i}<t\right)$ **then**12:        $\phi_{i}\leftarrow {\hat{X}}_{i}$13:        i←i+114:     **end if**15:    **end while**16:    Φ←sort(Φ)17:    $\psi_{s}\leftarrow median\left(\Phi \right)$18:    M←M−Φ19:    s←s+120:   **end while**21:**end procedure**

### 3.3. Deep Network

Our proposed deep neural network architecture consists of an input layer, two hidden layers (*h* and $h^{\prime }$) and an output layer; all these layers are fully connected to each other. To design the deep neural network architecture, we conducted several experiments with varying numbers of hidden layers, as well as varying numbers of neuron units within each hidden layer. We empirically concluded that when we increase the number of hidden layers beyond two, the performance decreases. We report and discuss all these results in detail in Section 4.

#### Implementation Details

We input the joint features extracted from motion sequences to our neural network. Each node of the first hidden layer *h* takes real-valued numbers, computes the weighted sum, and applies a non-linear activation function (sigmoid function) in order to execute the output as

(1) $$h_{p}=\frac{1}{1+e^{-\left[\sum_{j=0}^{J\times 3}x_{j}w_{j,p}\right]}}\;\mathrm{with}\;p\in \left\{1,2,3,\ldots ,P\right\},$$

where *P* is the total number of nodes for the first hidden layer. Similarly, at each unit of the second hidden layer $h^{\prime }$, we compute the output as

(2) $$h_{q}^{\prime }=\frac{1}{1+e^{-\left[\sum_{p=0}^{P}h_{p}w_{p,q}^{\prime }\right]}}\;\mathrm{with}\;q\in \left\{1,2,3,\ldots ,Q\right\},$$

where *Q* is the number of units for the second hidden layer. Finally, for this multi-class classification problem, we employ the softmax function in the output layer in order to yield the probability of each class at each unit of the output layer:

(3) $$o_{r}=\frac{e_{r}^{\sum_{q=0}^{Q}h_{q}^{\prime }w_{q,r}^{\prime \prime }}}{\sum_{r}\cdot e_{r}^{\sum_{q=0}^{Q}h_{q}^{\prime }w_{q,r}^{\prime \prime }}}\;\mathrm{with}\;r\in \left\{1,2,3,\ldots ,R\right\},$$

where *R* is the number of units for the output layer. The softmax function basically squashes a vector into the range of 0–1, and the sum of all the resulting elements is necessarily equivalent to 1. We exploit a cross-entropy cost function with the predicted value $o_{r}$ and the target value $t_{r}$ for this multi-nominal classification problem, e.g., the one-hot encoded vector $t_{r}=\left[0,0,0,1,0,\ldots ,R\right]$ contains just a single 1 at the 4th position. This cross-entropy cost,

(4) $$E=-\sum_{r}t_{r}\ln\left(o_{r}\right),$$

is computed at the output layer, and the errors are back-propagated towards the hidden layers in order to update the weight vectors w, $w^{\prime }$, and $w^{\prime \prime }$ with a gradient descent algorithm. For the implementation of the gradient descent, the derivative of the error *E* is computed with respect to each weight $w_{q,r}^{\prime \prime }$ that connects the hidden layer $h^{\prime }$ to the output layer with the softmax function,

(5) $$\frac{\partial E}{\partial w_{q,r}^{\prime \prime }}=\frac{\partial E}{\partial o_{r}}\cdot \frac{\partial o_{r}}{\partial w_{q,r}^{\prime \prime }}.$$

For each unit in the output layer *o* indexed by *r*, the gradients are

(6) $$\delta_{r}^{\prime \prime }=\frac{\partial E}{\partial o_{r}}=\left(o_{r}-t_{r}\right),$$

and the gradient with respect to $w_{q,r}^{\prime \prime }$ becomes

(7) $$\frac{\partial E}{\partial w_{q,r}^{\prime \prime }}=h_{q}^{\prime }\left(o_{r}-t_{r}\right).$$

Similarly, the derivative of the error *E* with respect to each weight $w_{p,q}^{\prime }$ that connects the first hidden layer *h* to the second hidden layer $h^{\prime }$ with the sigmoid function is

(8) $$\frac{\partial E}{\partial w_{p,q}^{\prime }}=\frac{\partial E}{\partial h_{q}^{\prime }}\cdot \frac{\partial h_{q}^{\prime }}{\partial w_{p,q}^{\prime }}.$$

For each unit in the hidden layer $h^{\prime }$ indexed by *q*, the gradients of the loss function are

(9) $$\delta_{q}^{\prime }=\frac{\partial E}{\partial h_{q}^{\prime }}=h_{q}^{\prime }\left(1-h_{q}^{\prime }\right)\cdot \sum_{r}\left(o_{r}-t_{r}\right)\cdot \left(w_{q,r}^{\prime \prime }\right)$$

and, with respect to weight $w_{p,q}^{\prime }$,

(10) $$\frac{\partial E}{\partial w_{p,q}^{\prime }}=h_{q}^{\prime }\left(1-h_{q}^{\prime }\right)\cdot \sum_{r}\left(o_{r}-t_{r}\right)\cdot \left(w_{q,r}^{\prime \prime }\right)\cdot \left(h_{p}\right).$$

The derivative of the error *E* with respect to each weight $w_{j,p}$ that connects the input layer to the first hidden layer *h* with the sigmoid function is

(11) $$\frac{\partial E}{\partial w_{j,p}}=\frac{\partial E}{\partial h_{p}}\cdot \frac{\partial h_{p}}{\partial w_{j,p}}.$$

For each unit in the first hidden layer *h* indexed by *p*, the gradients are

(12) $$\delta_{p}=\frac{\partial E}{\partial h_{p}}=h_{p}\left(1-h_{p}\right)\cdot \sum_{q}\delta_{q}^{\prime }\cdot \left(w_{p,q}^{\prime }\right)$$

and, with respect to weight $w_{j,p}$,

(13) $$\frac{\partial E}{\partial w_{j,p}}=h_{p}\left(1-h_{p}\right)\cdot \sum_{q}\delta_{q}^{\prime }\cdot \left(w_{p,q}^{\prime }\right)\cdot \left(x_{j}\right).$$

*Weight Updates:* The weights $w_{j,p}$ that establish the connection between the input and first hidden layers are updated as

(14) $$w_{j,p}=w_{j,p}+\eta \delta_{p}x_{j},$$

and similarly, the weights $w_{p,q}^{\prime }$ and weights $w_{q,r}^{\prime \prime }$ are updated as

(15) $$w_{p,q}^{\prime }=w_{p,q}^{\prime }+\eta \delta_{q}^{\prime }h_{p},$$

(16) $$w_{q,r}^{\prime \prime }=w_{q,r}^{\prime \prime }+\eta \delta_{r}^{\prime \prime }h_{q},$$

where η is the learning rate, which is kept equal to 0.01. We fix the maximum number of epochs to 1000, and the minimum performance gradient is kept at $1e^{-6}$. The training process stops if the validation performance deteriorates continuously for 5 consecutive epochs.

### 3.4. Action Score

To this end, we define the deep network architecture, and as an input, we provide the extracted features from the keyframe sequences of the motion in the form of joint positions to the network. Finally, in the last step, similar to [51,52], we calculate the *action score* frame by frame of the given keyframe sequence of the motion. On the basis of the probability determined through the deep network, we assign a vote to each keyframe involved in the given action sequences. We exploit the majority function here, where the majority count of the votes ultimately leads us to the prediction of the final action class.

## 4. Experiments

We evaluated our proposed approach extensively on a pool of benchmark MoCap datasets, namely CMU [33] and HDM05 [32], both of which are publicly available. We further categorized these datasets into four different types of datasets on the basis of action categories. The details about these datasets can be found in Section 4.1. We adopted a 5-fold cross-validation method in order to evaluate the performance of our proposed pipeline for classification. We performed a number of experiments in this context on these datasets. We first tuned the parameters that we utilized in our keyframe extraction algorithm and the deep network architecture for action recognition. We then started with the evaluation of our proposed keyframe extraction algorithm, as mentioned in Algorithm 1. Finally, we thoroughly evaluated the performance of our proposed framework by comparing it with other existing approaches.

### 4.1. Datasets

#### 4.1.1. HDM05 Dataset

HDM05 [32] is a well-defined popular dataset that contains almost 2337 sequences with 130 motion classes performed by 5 different actors. The Vicon MX system with 12 high-resolution cameras was used to capture the motions at a sampling rate of 120 Hz. The ultimate 3D skeleton consists of 31 joints in total. We categorized the HDM05 dataset [32] into two groups according to the number of classes, as found in the literature, as follows.

*HDM05-65:* As stated in [18], most motion classes can be combined into one distinctive major motion class; e.g., *shuffle2StepsLStart*, *shuffle2StepsRStart*, *shuffle4StepsLStart*, *shuffle4StepsRStart* belong to the motion category *shuffle*; thus, they are represented as one motion class, i.e., *shuffle*. As a result, we came up with 65 motion classes, which are the same as those described in [18,19].

*HDM05-14:* This dataset, extracted from HDM05, consists of 14 motion classes: *runOnPlace*, *shuffle*, *sneak*, *sitDownFloor*, *sitDownKnee*, *sitDownTable*, *skier*, *sitDownChair*, *squat*, *staircaseDown*, *staircaseUp*, *standUpSitChair*, *standUpLie*.

#### 4.1.2. CMU Dataset

Our second dataset is CMU [33], which is also considered a very popular dataset in the research community. The Vicon motion capture system, consisting of 12 infrared MX-40 cameras, recorded motions with a sampling rate of 120 Hz [33]. In the CMU dataset, the 3D skeleton also consists of 31 joints in total. We again categorized this dataset into two groups according to the number of classes as follows.

*CMU-30:* The dataset CMU-30 consists of 30 distinctive motion classes. It contains 278 labeled motion clips belonging to 30 different motion categories. A total of 33 different subjects participated in the recording of these motion clips, as mentioned in [37].

*CMU-14:* The dataset CMU-14 contains 14 motion classes: *cartwheel*, *jump*, *pick*, *swing*, *balanceWalk*, *walkBackwardOnToes*, *run*, *hopOnLeftFoo*t, *boxing*, *walk*, *getUpFromFloor*, *breastStroke*, *getUpFromChair*, *mickeyWalk*.

### 4.2. Parameters

We evaluated the impact of the parameters involved in our proposed framework on the overall performance of our approach. We first tuned these parameters and ultimately fixed their values for the other experiments.

#### 4.2.1. Threshold

**Figure 3:**
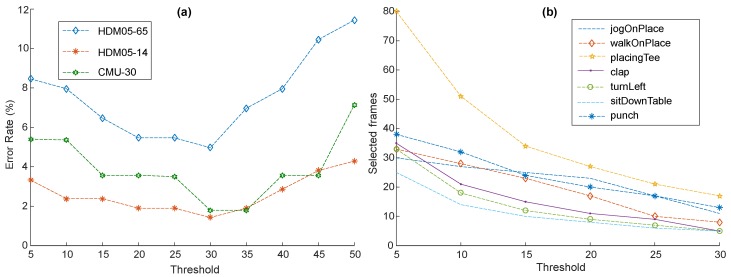
(**a**) Threshold tuning on different datasets. (**b**) The number of keyframes extracted through different threshold values for a variety of motions.

For the performance assessment of our proposed keyframe extraction technique, we first adjusted the threshold value empirically. From the experiments, we observed that as we increased the threshold value, the error decreased up to a certain point and then started increasing again. We fixed the threshold value to t=30 for all other experiments; at this threshold, the system obtained the best results, as is quite obvious in Figure 3a. We also conducted another experiment to see the impact of the threshold value on the selection of the keyframes, i.e., how many frames can be eliminated from the motion by the selection of the threshold. The compression ratio increased with the increase in the threshold. More precisely, the number of selected keyframes was reduced when the threshold had higher values, as shown in Figure 3b.

#### 4.2.2. Deep Network

We developed and compared various deep neural network configurations in order to tune the number of hidden layers, as well as the number of neurons within a hidden layer. We performed experiments with one, two and three hidden layers and with varying numbers of neurons within a hidden layer in the deep neural network architecture. The overall impact of using a different number of layers with a different number of neurons can be seen in Table 1. Although the results obtained with just one hidden layer are significant, with an accuracy of 93.53%, the highest accuracy (95.14%) was achieved by employing two hidden layers in the deep network for the process of action recognition. From the experiments, we empirically concluded that the performance in terms of accuracy decreased when more than two hidden layers were exploited in the deep neural network. Similarly, increasing or decreasing the number of neurons beyond 85 for the first hidden layer and 80 for the second hidden layer diminished the performance of the network, as well. As a result, we stopped going deeper and fixed the two hidden layers with 85 and 80 neurons, respectively, with the sigmoid activation function. For the output layer, we employed the softmax activation function in our proposed deep neural network architecture. The input provided to our deep network almost has low dimensionality (31×3), and the maximum action classes are just only 65 in total in case of HDM05 dataset; as a result, we obtained promising results with two hidden layers only.

### 4.3. Comparison with State-of-the-Art Methods

#### 4.3.1. Keyframes

We evaluated our proposed keyframe extraction approach by carrying out different types of experiments. We first examined how the keyframes affected the accuracy of action recognition. We performed a comparison between scenarios in which (i) we employed all the frames available in the motion category for the process of action recognition; (ii) only the keyframes extracted through our proposed Algorithm 1 were used in the process of action recognition; and (iii)–(iv) the keyframes were selected randomly with varying sizes: i.e., $\xi_{f}$, the number of keyframes with a size equal to the number of keyframes extracted through Algorithm 1, and $\xi_{f}^{\prime }$, the number of keyframes with a size that was double the number of keyframes extracted through Algorithm 1. Moreover, we adapted a 10-fold cross-validation procedure for the random selection of the keyframes. The results presented in Table 2 demonstrate that our proposed Algorithm 1 obtained the best results in terms of accuracy as compared with the other models mentioned above for different motion categories.

**Figure 4:**
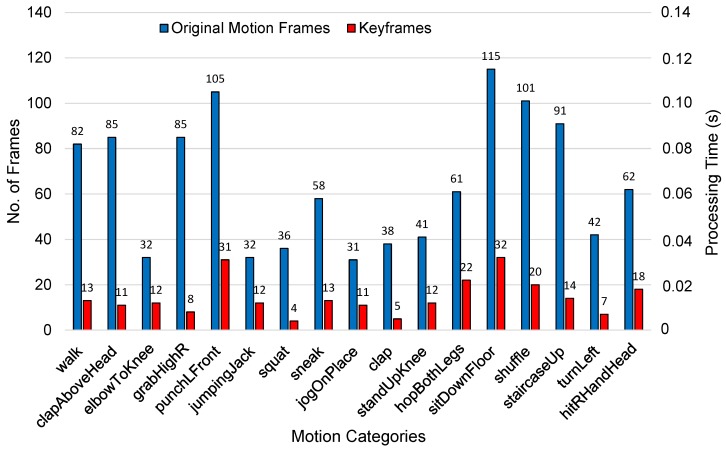
A comparison in terms of the number of frames between the keyframes extracted through Algorithm 1 against the original total number of frames for different types of motions. The processing time for the extraction of keyframes (seconds/frame) is also reported.

Our next experiment determined how many keyframes were extracted for a variety of motion categories and how much processing time was required for the extraction of these frames. The overall results for different motion categories are shown in Figure 4. For example, for the *walk* motion class, the number of frames was reduced from 82 to 13; similarly, for the motion class *sitDownFloor*, the number of frames was reduced from 115 to 32, etc. The processing time required for the extraction of each keyframe was approximately 0.062 seconds. Further details about the processing time are available in Section 4.3.3.

**Figure 5:**
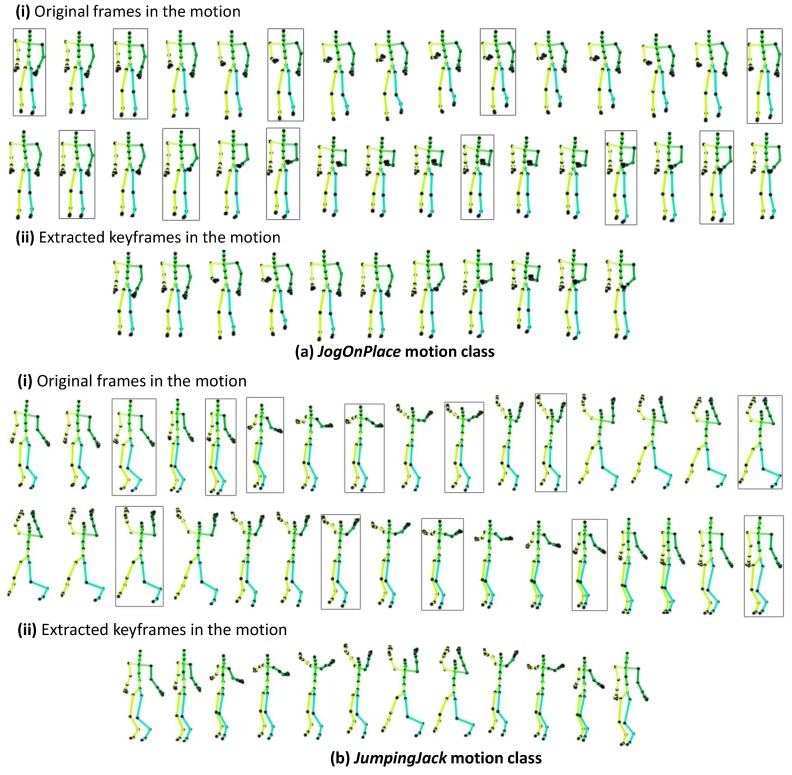
Qualitative results for our proposed keyframe extraction Algorithm 1 for the motion categories (**a**) *jogOnPlace* and (**b**) *jumpingJack*. The extracted keyframes are shown in boxes and also visualized separately to show a clear view.

We also assessed our keyframe extraction approach qualitatively, and we show the results in Figure 5, where the actual frames in the motion class and the keyframes extracted through our proposed Algorithm 1 are visualized. For example, there are 31 original frames in the motion category *jogOnPlace* and 32 frames in the *jumpingJack* motion; for the two motion classes, 11 and 12 keyframes were extracted, respectively, which have most of the information about the motion, as evident in Figure 5.

From these experimental results, we conclude that extracting a few informative frames through the proposed keyframe method, rather than using all the frames of a motion sequence, is sufficient to recognize the action accurately. Moreover, our keyframe extraction approach improves the accuracy of action recognition by using a few informative frames rather than exhausting all the existing frames of the motions.

#### 4.3.2. Action Recognition

We finally evaluated our proposed framework for action recognition and performed extensive experiments on the benchmark MoCap datasets CMU [33] and HDM05 [32], which were further categorized into four groups of datasets, HDM05-65, HDM05-14, CMU-30, and CMU-14, as mentioned in Section 4.1. We discuss all the results separately in detail as follows.

##### Evaluation on HDM05-65

In the case of the HDM05-65 dataset with 65 motion classes, we followed the same experimental protocol as that proposed in [18,19]. For this dataset, our approach achieved competitive results, with 95.14% accuracy, in comparison with other techniques. Although our approach did not outperform comparatively, it still produced competitive and very promising results. Our approach with deep neural network architecture performed better as compared with other popular classifiers, including Support Vector Machine (SVM), Multi-layer perceptron (MLP), Extreme Learning Machine [18], Convolutional Neural Network (CNN) [1], Deep Bidirectional Recurrent Neural Network (DBRNN-T) [19] with the *tanh* activation function, and Deep Unidirectional Recurrent Neural Network (DBRNN-T) [19] with the *tanh* activation function on the same HDM05-65 dataset. The Hierarchical Bidirectional Recurrent Neural Network (HBRNN-L) [19] with Long-Short Term Memory (LSTM) had the highest accuracy. Other RNN variants, such as the Hierarchical Unidirectional Recurrent Neural Network (HURNN-L) [19] with LSTM, Deep Bidirectional Recurrent Neural Network (DBRNN-L) [19] with LSTM, and Deep Unidirectional Recurrent Neural Network (DURNN-L) [19] with LSTM, also produced results with high accuracies, but there was very little marginal difference in accuracy in comparison with our proposed approach, as observed in Table 3. Moreover, our proposed deep neural network architecture is a comparatively simple architecture with less complexity. On the other hand, all the different categories of recurrent neural networks proposed in [19] consist of a large network structure with feedback connections, where the previous layer with a node influences itself to form a loop and ultimately leads towards high complexity.

**Figure 6:**
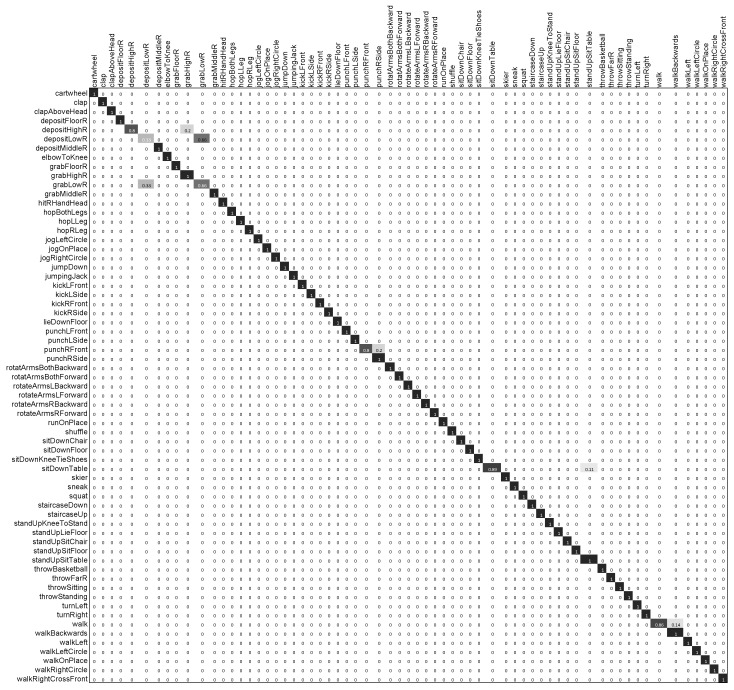
Confusion matrix of our proposed approach on the HDM05-65 dataset.

The confusion matrix for HDM05-65, presented in Figure 6, shows that our proposed framework performed well on almost all types of motion. The few motion categories for which our approach did not perform significantly well are the *depositLow* and *grabLow* motions. Since both of these motion categories are very similar to each other, our approach misclassified them: e.g., the motion class *grabLow* was 33% misclassified as the motion *depositLow*, and alternatively, *depositLow* was 66% misclassified as the *grabLow* motion. As a result, the overall performance of our scheme decreased to 95.14% accuracy.

##### Evaluation on HDM05-14

**Figure 7:**
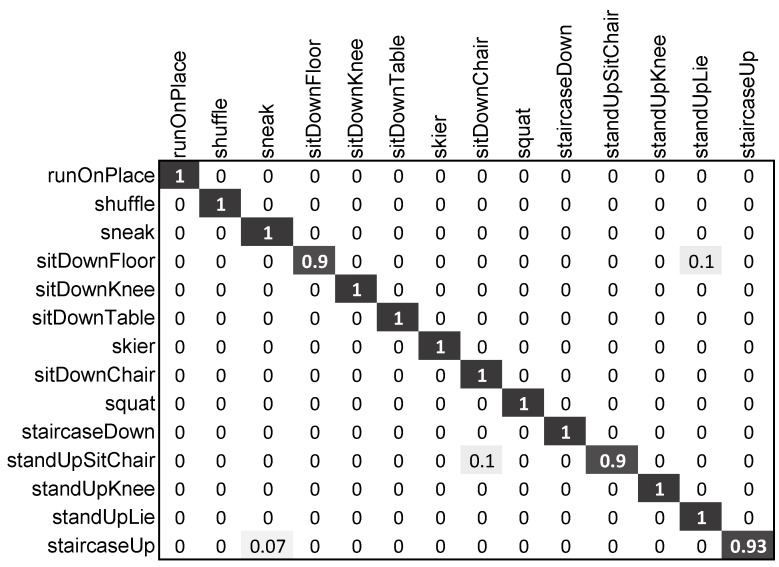
Confusion matrix of our proposed approach on the HDM05-14 dataset.

On the dataset HDM05-14 with 14 motion classes, our proposed scheme outperformed the other existing state-of-the-art approaches [1,62], and it achieved 98.6% accuracy. The addition of keyframes with Normalized Trajectories (NT) substantially contributed to the improved performance of our proposed framework, as is obvious in Table 3. The detailed version of the results of our approach on the HDM05-14 dataset in the form of a confusion matrix is presented in Figure 7. Although some of the actions were misclassified by our approach (*sitDownFloor* and *standUpSitChair* were misclassified; *staircaseUp* was misclassified as *steak*), our approach still performed significantly well.

##### Evaluation on CMU-30

On the CMU-30 dataset, the results of our approach are outstanding as compared with other state-of-the-art methods (see Table 3). Although the method of two-step Support Vector Machine (SVM) fusion with Tree Structure Vector Quantized (TSVQ) [37] had the best results, our approach provided competitive results with an accuracy of 99.3% and outperformed other approaches, such as the Similarity Suffix Array, Pose-Histogram Classifier, and Two-Step Score Fusion [37]. All these results can be seen in detail in Table 4, where it is quite obvious that our approach only misclassified the *bouncyWalk* motion and achieved 99.3% accuracy, while the method of two-step SVM fusion with TSVQ [37] also misclassified the motion *rhymeTeaPot* and achieved 99.6% accuracy.

**Figure 8:**
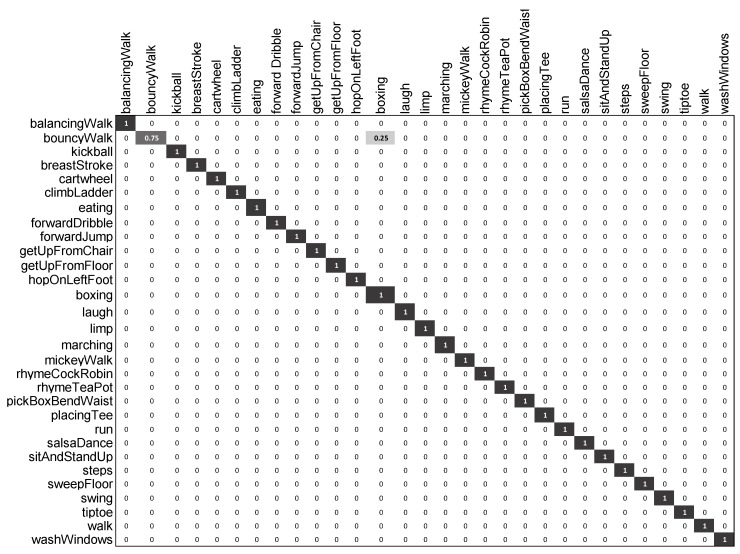
Confusion matrix of our proposed approach on the CMU-30 dataset.

The confusion matrix in Figure 8 provides a detailed description of the results of our approach on the CMU-30 dataset. All the motion classes were correctly classified except *bouncyWalk*, which was misclassified as the motion *boxing*.

##### Evaluation on CMU-14

**Figure 9:**
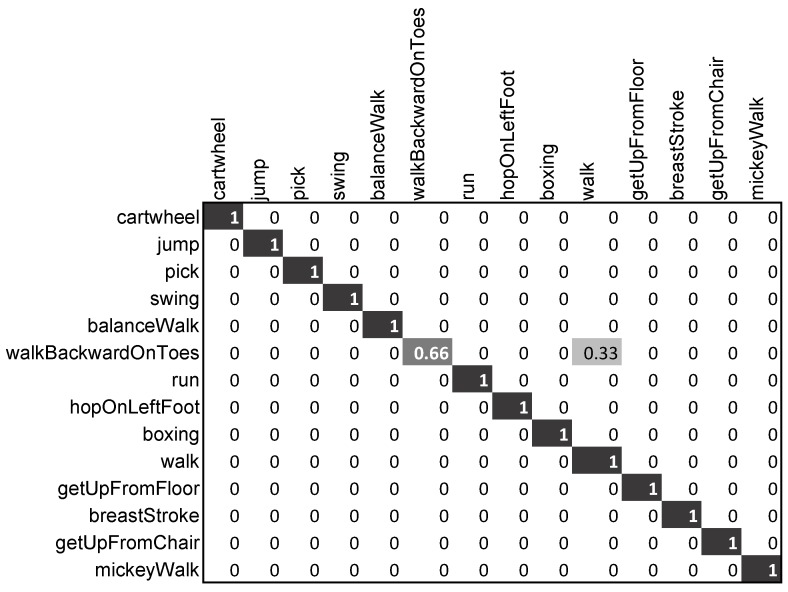
Confusion matrix of our proposed approach on the CMU-14 dataset.

On the CMU-14 MoCap dataset, we compared our approach with [44,48]. These methods use the CMU MoCap dataset, as well as their own dataset with 14 classes, recorded by utilizing a Vicon (http://www.vicon.com/) motion capture device, while we conducted experiments on only the CMU-14 MoCap dataset. Our proposed scheme outperformed both approaches in [44,48] and obtained 98.5% accuracy on the CMU-14 dataset. In this case, we employed 85% of the training data for training purposes. From the detailed analysis presented in Table 3 and in Figure 9, we observe that out of 14 motion classes, only one motion class, *walkBackwardsOnToes*, was misclassified as the *walk* motion category.

#### 4.3.3. Processing Time

We conducted our experiments on a Core I7 with 8GB RAM and a Windows operating system. The proposed keyframe extraction methods took approximately 0.062 seconds to extract keyframes from a motion of 60 frames. The input query motion took roughly 0.0085 seconds per frame for action recognition. More precisely, the overall time for feature extraction, as well as action recognition, was ∼0.0095 seconds per frame.

### 4.4. Discussion

We evaluated our proposed method extensively on different types and sizes of MoCap datasets. From the experimental results described in Table 3 and Table 4, our proposed algorithm performs comparatively well. We observe that our approach not only classifies the *distinct* action classes very accurately, e.g., *cartwheel*, *walk*, *jumpingJack*, and *clap*, but also correctly classifies most of the *similar* action classes, e.g., *turnLeft* vs. *turnRight*, *jogLeftCircle* vs. *jogRightCircle*, *jogOnPlace* vs. *runOnPlace*, *walkLeftCircle* vs. *walkRightCircle*, *kickLSide* vs. *kickRSide* and *punchLFront* vs. *punchRFront*. The few motion categories that are misclassified are *immensely similar* to each other: *depositHighR* is misclassified as *grabHighR*, and *depositLowR* is misclassified as *grabLowR*. In a few cases in which the action is the combination of a sequence of atomic movements that are further shared among different classes, our keyframe-based approach might mislead: e.g., *sitDownTable* is misclassified as *standUpSitTable*, and the majority of the keyframes extracted from *standUpSitTable* belong to the subclass *SitTable*, and, as a result, the *sitDownTable* class is predicted. Short, single actions with multiple classes, such as *standUpSitTable*, may create ambiguity for our proposed method.

Our proposed approach is data-driven, and it performs well enough when at least a few similar action poses of the input action are available in the MoCap dataset; the performance may deteriorate when a similar input motion is not available in the MoCap dataset. Our proposed method is fast enough with a short latency (∼0.0095 seconds per frame), which is a crucial requirement for real-time online systems. Because our approach is keyframe-dependent, it has the capacity to compensate for missing information, as well.

## 5. Conclusions

In this paper, we present a novel action recognition schema that relies on keyframes extracted from action sequences. The extracted keyframes enhance the process by providing information that is free from redundancy but carries the most relevant details about the action that exists in the motion. We conducted a variety of experiments to evaluate the keyframe extraction results, and we concluded that a few but significant information-carrying frames, rather than all frames with redundant information, are sufficient. We started with the normalization of 3D poses and extract joint features, on the basis of which we constituted our proposed deep neural network. We empirically configured the number of hidden layers, as well as the number of units within a hidden layer, by carrying out several experiments. We conclude from these experiments that, in our case, two hidden layers with 85 and 80 neurons are adequate to get substantial results. Finally, for thorough and detailed performance evaluations, we worked on four different datasets with varying numbers of motion categories, i.e., HDM05-65, HDM05-14, CMU-30, and CMU-14. On the HDM05 dataset, our proposed framework comparatively produced very competitive results, with 95.14% accuracy on HDM05-65 and 98.6% accuracy on the HDM05-14 dataset. On the CMU dataset, our proposed approach outperformed other techniques, with 98.5% accuracy on the CMU-14 dataset and 99.3% accuracy on the CMU-30 dataset. Furthermore, our approach has satisfactory efficiency in terms of time, as well. It takes roughly 0.0095 seconds per frame to recognize the action.

In future work, the proposed framework may be extended to perform action recognition and motion segmentation simultaneously. Furthermore, the proposed method for action classification can be coupled with gait analysis and person identification. Another important direction might be the integration of action recognition and pose estimation together in 3D-3D, 2D-3D, and 3D-2D scenarios. To date, we have worked only with action classes that are performed by a single person, and this approach can be extended to multiple persons interacting with each other.

## Figures and Tables

**Table 1:** Deep neural network performance with varying numbers of neurons within a hidden layer, as well as with different numbers of hidden layers.

Input Layer	Hidden Layer 1	Hidden Layer 2	Hidden Layer 3	Output Layer	Accuracy (%)
93	75	-	-	65	89.55%
93	100	-	-	65	90.54%
93	125	-	-	65	91.04%
93	150	-	-	65	92.53%
93	175	-	-	65	92.53%
93	200	-	-	65	93.03%
93	225	-	-	65	93.53%
93	250	-	-	65	93.53%
93	275	-	-	65	93.03%
93	300	-	-	65	93.03%
93	75	75	-	65	91.54%
93	75	80	-	65	91.54%
93	75	85	-	65	92.04%
93	75	90	-	65	92.04%
93	80	75	-	65	92.04%
93	80	80	-	65	93.53%
93	80	85	-	65	93.53%
93	80	90	-	65	93.03%
93	85	75	-	65	92.53%
**93**	**85**	**80**	-	**65**	**95.14%**
93	85	85	-	65	93.53%
93	85	90	-	65	93.53%
93	90	75	-	65	92.04%
93	90	80	-	65	94.53%
93	90	85	-	65	92.53%
93	90	90	-	65	92.04%
93	85	80	75	65	93.53%
93	85	80	80	65	94.03%
93	85	80	85	65	94.03%
93	85	80	90	65	93.53%

**Table 2:** A comparison between scenarios in which (i) all the original frames in the specified motion are utilized in the process of action recognition, (ii) only the keyframes extracted through Algorithm 1 are employed in the process of action recognition, (iii) $\xi_{f}$ keyframes selected randomly are used, and (iv) $\xi_{f}^{\prime }$ keyframes selected randomly are used.

Motions	(i) All Frames		(ii) Keyframes		(iii) $\left(\xi_{f}\right)$		(iv) $\left(\xi_{f}^{\prime }\right)$	
	Frames	Acc.	Frames	Acc.	Frames	Acc.	Frames	Acc.
standUpLie	327	50%	93	100%	93	40%	186	50%
lieDownFloor	277	50%	81	100%	81	50%	162	50%
throwStandingHighR	239	66.66%	98	100%	98	69.97%	196	66.66%
standUpSitChair	172	50%	31	100%	31	55%	62	50%
grabMiddleR	151	66.66%	35	100%	35	59.99%	70	69.94%
depositMiddleR	142	100%	20	100%	20	70%	40	100%
sitDownTable	117	50%	11	100%	11	55%	22	40%
standUpSitTable	177	100%	24	100%	24	90%	48	80%
grabLowR	186	66.66%	47	66.66%	47	43.32%	94	39.97%
turnLeft	145	100%	29	100%	29	79.99%	58	100%
punchLFront	226	100%	77	100%	77	85%	154	97.75%
sitDownFloor	159	100%	54	100%	54	75%	108	100%
depositLowR	224	33.33%	52	33.33%	52	33.33%	104	33.33%
squat	536	100%	89	100%	89	98.57%	178	100%
elbowToKnee	409	100%	162	100%	162	100%	324	100%

**Table 3:** Comparison with other state-of-the-art methods on the datasets HDM05-65, HDM05-14, CMU-30, and CMU-14. The results are presented in top-down order. The results using our approach are shown in **bold** text. * The CMU dataset with 14 classes was used, as well as the dataset recorded with their own Vicon (http://www.vicon.com/) motion capture device. ***Note:*** Normalized Trajectories (NT), relative positions of joints (PO), Temporal Differences (TD), Tree Structure Vector Quantized (TSVQ), Hierarchically Bidirectional Recurrent Neural Network with Long-Short Term Memory (LSTM) (HBRNN-L). For more details, see Section 2 and Section 4.3.2.

Dataset	Approach	Algorithm	Features	Training	Accuracy
HDM05-65	Du et al. [19]	HBRNN-L	NT	90%	96.90%
		HURNN-L	NT		96.70%
		DBRNN-L	NT		96.70%
		DURNN-L	NT		96.62%
	Cho et al. [18]	Hybrid MLP (λ=0.5)	PO+TD		95.59%
		Hybrid MLP (λ=0.9)	PO+TD		95.55%
		Hybrid MLP (λ=0.1)	PO+TD		95.46%
		Hybrid MLP (λ=0.1)	PO+TD+NT		95.21%
		MLP	PO+TD		95.20%
	**Our approach**	**DNN**	**NT+Keyframes**		95.14%
	Cho et al. [18]	SVM	PO+TD+NT		95.12%
		Hybrid MLP (λ=0.9)	PO+TD+NT		95.04%
		SVM	PO+TD		94.95%
		MLP	PO+TD+NT		94.86%
		Hybrid MLP (λ=0.5)	PO+TD+NT		94.82%
	Du et al. [19]	DBRNN-T	NT		94.79%
		DURNN-T	NT		94.63%
	Sedmidubsky et al. [1]	CNN+KNN	NT		93.9%
	Cho et al. [18]	ELM	PO+TD+NT		92.76%
		ELM	PO+TD		91.57%
HDM05-14	**Our approach**	**DNN**	**NT+Keyframes**	50%	98.6%
	Sedmidubsky et al. [1]	CNN+KNN	NT		94.3%
	Elias et al. [62]	CNN+KNN	NT		87.4%
CMU-30	Kadu and Kuo [37]	Two-Step SVM Fusion	TSVQ	80%	99.6%
	**Our approach**	**DNN**	**NT+Keyframes**		99.3%
	Kadu and Kuo [37]	Two-Step Score Fusion	TSVQ		98.2%
		Pose-Histogram Classifier	B-PL04		95.6%
			B-PL06		95.6%
		Motion-String Similarity	A-SL12		95.6%
			A-SL13		95.6%
		Pose-Histogram Classifier	B-PL05		95.3%
			B-PL03		92.8%
		Motion-String Similarity	A-ML12		82.3%
			A-ML13		80.5%
CMU-14	**Our approach**	**DNN**	**NT+Keyframes**	85%	98.5%
	Wu et al. [44]*	Hierarchical Tree	3D Trajectories		98.1%
	Wu et al. [48]*	Smith–Waterman	3D Trajectories		97.0%

**Table 4:** A detailed comparison between our approach and other state-of-the-art methods for all 30 motion classes on the CMU-30 dataset. Methods A-ML12 and A-ML13 refer to Max-parameter with Levels 12 and 13, while methods A-SL12 and A-SL13 refer to Sim-parameter with Levels 12 and 13 [37]. Methods B-PL03, B-PL04, B-PL05, and B-PL06 are the Pose histogram with Levels 03, 04, 05, and 06, respectively [37]. Methods C and D refer to Two-Step Score Fusion and Two-Step Support Vector Machine (SVM) Fusion, respectively [37].

Motion Categories	A-ML12	A-ML13	A-SL12	A-SL13	B-PL03	B-PL04	B-PL05	B-PL06	(C)	(D)	Ours
run(27)	96%	96%	100%	96%	96%	100%	96%	96%	100%	100%	100%
walk(47)	85%	85%	97%	100%	97%	97%	97%	97%	100%	100%	100%
forwardJump(9)	88%	88%	88%	100%	88%	88%	77%	100%	100%	100%	100%
forwardDribble(5)	100%	100%	100%	100%	100%	100%	100%	100%	100%	100%	100%
cartWheel(5)	100%	100%	100%	100%	100%	100%	100%	100%	100%	100%	100%
kickball(6)	100%	100%	100%	100%	83%	83%	83%	83%	100%	100%	100%
boxing(7)	0%	0%	85%	100%	100%	100%	100%	100%	100%	100%	100%
mickeyWalk(7)	100%	100%	100%	100%	85%	100%	100%	100%	100%	100%	100%
sitAndStandUp(5)	80%	80%	100%	100%	100%	100%	100%	80%	100%	100%	100%
laugh(6)	66%	100%	100%	100%	100%	100%	100%	100%	100%	100%	100%
sweepFloor(5)	40%	40%	100%	100%	80%	100%	100%	100%	100%	100%	100%
washWindows(5)	640%	60%	100%	100%	100%	100%	100%	100%	100%	100%	100%
climbLadder(5)	100%	100%	100%	100%	100%	100%	100%	100%	100%	100%	100%
steps(7)	57%	85%	100%	100%	100%	100%	100%	100%	100%	100%	100%
eating(5)	100%	100%	100%	100%	100%	100%	100%	100%	100%	100%	100%
tiptoe(5)	100%	60%	100%	100%	100%	100%	100%	100%	100%	100%	100%
pickBoxBendWaist(6)	100%	100%	83%	100%	83%	83%	100%	100%	100%	100%	100%
limp(5)	100%	80%	100%	100%	100%	100%	100%	100%	100%	100%	100%
balancingWalk(12)	83%	75%	100%	100%	83%	100%	91%	91%	100%	100%	100%
getUpFromChair(5)	80%	80%	100%	80%	80%	100%	100%	60%	100%	100%	100%
breastStroke(6)	50%	16%	83%	83%	100%	100%	83%	83%	83%	100%	100%
hopOnLeftFoot(6)	66%	100%	100%	100%	100%	83%	100%	100%	100%	100%	100%
bouncyWalk(6)	66%	66%	100%	100%	50%	66%	83%	100%	100%	100%	75%
marching(10)	100%	100%	100%	90%	100%	100%	100%	100%	100%	100%	100%
rhymeTeaPot(16)	81%	81%	75%	75%	75%	87%	87%	87%	75%	93%	100%
rhymeCockRobin(10)	60%	40%	86%	86%	93%	86%	80%	100%	100%	100%	100%
swing(10)	100%	100%	90%	100%	100%	100%	100%	100%	100%	100%	100%
placingTee(5)	100%	100%	100%	80%	80%	80%	100%	80%	100%	100%	100%
salsaDance(15)	86%	73%	100%	95%	100%	100%	100%	100%	100%	100%	100%
getUpFromFloor(10)	80%	80%	100%	100%	80%	100%	100%	80%	100%	100%	100%
**Total(278)**	82.3%	80.5%	95.6%	95.6%	92.8%	95.6%	95.3%	95.6%	98.2%	99.6%	99.3%
